# Applying Nanofiltration to Decrease Energy Consumption and Sensitivity toward Feed Composition Fluctuations in Salt Production

**DOI:** 10.3390/membranes14050103

**Published:** 2024-04-29

**Authors:** Marian Turek, Krzysztof Mitko, Paweł Skóra

**Affiliations:** Department of Inorganic, Analytical Chemistry and Electrochemistry, Faculty of Chemistry, Silesian University of Technology, ul. B. Krzywoustego 6, 44-122 Gliwice, Poland; krzysztof.mitko@polsl.pl (K.M.); pawel.skora@polsl.pl (P.S.)

**Keywords:** nanofiltration, coal mine water, evaporated salt production

## Abstract

The only currently active industrial-scale plant that uses coal mine brines, located in Czerwionka-Leszczyny, uses ZOD (Zakład Odsalania Dębieńsko, the name of the plant’s former owner) technology, based on mechanical vapor compression evaporators. The plant produces evaporated salt that meets the specifications for edible salt; however, the technology is highly energy-consuming. The presented work focuses on the modeling of ZOD technology if applied to the water treatment of the ‘Ziemowit-650’ coal mine. Using the results of bench-scale investigation of brine nanofiltration and a mathematical model of ZOD technology based on Czerwionka-Leszczyny performance, the energy consumption per ton of produced salt was estimated for two cases: (1) ZOD technology treating the ‘Ziemowit-650’ brine and (2) ZOD technology treating the permeate of nanofiltration (NF) working on the ‘Ziemowit-650’ brine. The sensitivity of the system was investigated in the range of −10% to + 10% of Cl^−^, SO_4_^2−^, Mg^2+^, and Ca^2+^ concentration, assuming that the sodium concentration also changes to meet the electroneutrality requirement. The results show that nanofiltration pretreatment not only decreases energy consumption but it also makes salt production less sensitive to fluctuations in feed water composition.

## 1. Introduction

Membrane technologies are widely used to mitigate problems related to saline wastewater discharge from various branches of industry. One of the industries that implements membrane-based technologies is coal mining. Despite the fact that coal mining is being phased out in favor of alternative and cleaner sources of energy, its environmental effects will not disappear anytime soon. Poland produces 95% of the hard coal in the European Union, with 80.33% of documented hard coal resources in the Upper Silesian Coal Basin (UCSB) [1]. The dewatering of coal mines is the main cause of disturbances in the hydrochemical regime of the USCB. Mine water discharges have an important influence on its surface water quality and quantity. The UCSB is located hundreds of kilometers from the nearest sea, so discharge to inland surface waters is the only viable option. The waters of these mines are generally discharged into tributaries of the upper Vistula and upper Odra rivers [2], where they affect surface water both quantitatively and qualitatively, especially in small streams where significant changes in the hydrological regime are caused by large loads of contaminants in the mine discharges [3].

One of the possible solutions for the discharge of industrial wastewater is resource recovery and in the case of saline effluents, the two main recoverable products are demineralized water and sodium chloride. In Poland, there is only one active industrial-scale plant that uses coal mine brines for salt production. The plant is located in Czerwionka-Leszczyny and uses ZOD technology (named after Zakład Odsalania Dębieńsko, a company that owned the plant before 2015 when it was acquired by the current owner) [4,5,6], which is based on mechanical vapor compression evaporators. The plant, which receives the waters of the ‘Budryk’ coal mine, produces evaporated salt that complies with the specifications of edible salt; however, the technology is highly energy-consuming, which limits its replication in the coal mining industry. One of the ways to address the issues inherent in ZOD technology is by applying nanofiltration (NF) as a pretreatment before ZOD technology. The nanofiltration pretreatment can be advantageous in this use case because it can be retrofitted to the existing plant, without the costly modifications of downstream treatment.

Nanofiltration, due to its high rejection of all ions except monovalent ones [7], is widely used to eliminate the hardness of groundwater [8] or recover important substances such as proteins and sugars. NF can also be an alternative to reverse osmosis (RO) for the desalination of brackish waters, where SO_4_^2−^ is the dominant anion [9]. Nanofiltration is used as a pretreatment [10] before other methods such as reverse osmosis, as well as for the treatment of various mine waters, including acid mine waters [11,12,13] and discharge from oil sand mining [14]. Nanofiltration has also been used as a unit operation preceding thermal methods. For example, nanofiltration was proposed before the multi-effect distillation in a trigeneration system, which resulted in improved performance of the desalination system [15]. Nanofiltration can also be employed before the multi-stage flash distillation to avoid calcium sulfate scale on the heat exchange surface [16] or as a deaeration step [17]. An integrated nanofiltration/precipitation/multi-effect distillation system was also employed for the recycling of spent brines from ion-exchange column regeneration [18]. Another possibility is to use an integrated precipitation/nanofiltration/multi-effect distillation system to pre-concentrate spent desalination brines before recovering valuable critical materials [19].

Nanofiltration has previously been investigated as a method to increase salt recovery in the production of evaporated salt using coal mine waters as feed. Two-pass nanofiltration with intermediate crystallization of gypsum was found to increase salt recovery and decrease energy consumption when using ‘Budryk’ coal mine water [20]. The effect of two-pass nanofiltration on the desalination of ‘Budryk’ coal mine water was also tested on a pilot scale [21].

**Figure 1:**
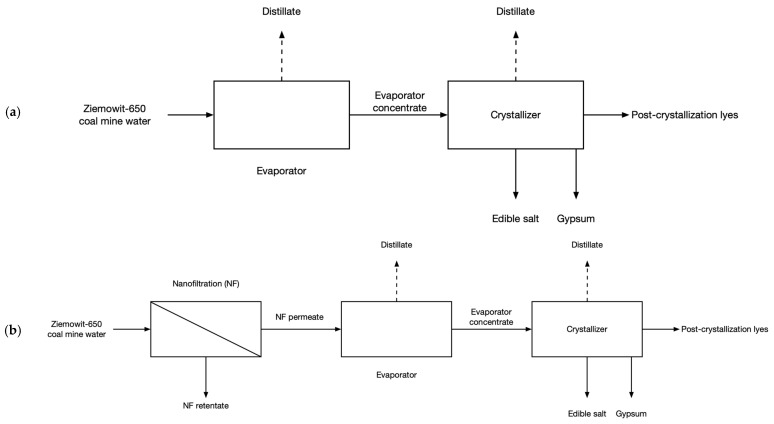
Flowchart of the discussed system: (**a**) ZOD technology and (**b**) ZOD technology with NF pretreatment.

The presented work focuses on the modeling of ZOD technology if applied for the treatment of the alternative feed stream, the ‘Ziemowit-650’ coal mine water (TDS of 82.6 g/dm^3^), which can be a prospective alternative to ‘Budryk’ water (TDS of 30.8 g/dm^3^ for the less saline part of ‘Budryk’ water) as it is more saline. This increased salinity makes it impossible to concentrate coal mine water using reverse osmosis because of the very high osmotic pressure but high-pressure nanofiltration can still be used. Using the results of the bench-scale investigation of brine nanofiltration and a mathematical model of ZOD technology based on the performance of Czerwionka-Leszczyny, the energy consumption per ton of salt produced was estimated for two cases (see Figure 1): (a) ZOD technology treating the ‘Ziemowit-650’ coal mine water and (b) ZOD technology treating the permeate of nanofiltration (NF) working on the ‘Ziemowit-650’ coal mine water.

## 2. Experimental

### 2.1. Batch-Mode Nanofiltration

Nanofiltration was carried out using the dead-end Sterlitech^®^ HP 4750X stirred cell module (Sterlitech Corporation, Auburn, WA, USA) with an effective membrane area of 14.6 cm^2^. A sample of ‘Ziemowit-650’ coal mine water pretreated with ultrafiltration was used. Table 1 presents the concentration of the main ionic species present in the coal mine water (Cl^−^, Ca^2+^, Mg^2+^, SO_4_^2−^, and Na^+^), as well as the concentration of barium and strontium, two micro-elements that were included in the scaling risk analysis. A commercial nanofiltration flat-sheet membrane Synder Filtration^TM^ NFW (Synder Filtration, Vacaville, CA, USA) was tested (see Table 2). All experiments were carried out at room temperature and 40 bar pressure. A high stirring speed (1200 rpm) was applied to avoid concentration polarization. The experimental protocol was as follows: (1) fill the module with 300 mL of ‘Ziemowit-650’ coal mine water; (2) run nanofiltration at 40 bar until 30 mL of permeate is collected (10% of recovery) to compact the membrane and equilibrate it with the feed water; (3) recycle the collected permeate back to the module; and (4) run nanofiltration and collect every 30 mL of permeate (10%) as separate samples. Nanofiltration permeate samples were collected during the experiment. The concentration of Cl^−^ was determined using argentometric titration using Mohr’s method [22], whereas the concentration of Mg^2+^, Ca^2+^, SO_4_^2−^, Ba, and Sr were determined using inductively coupled plasma atomic emission spectrometry (ICP-AES) on a Varian 710-ES spectrometer (Varian, Belrose, Australia) equipped with a OneNeb nebulizer (Agilent, Santa Clara, CA, USA) and a double pass glass cyclonic spray chamber. The residual retentate was also analyzed.

### 2.2. Scaling Risk Analysis

The possibility of scaling on the nanofiltration membrane surface was assessed based on the ionic composition of the retentate obtained in the batch-mode experiment. To calculate the saturation of sparingly soluble salts, PHREEQC Version 3 geochemical modeling software was used in conjunction with the Pitzer database, assuming a pH of 3, a temperature of 25 °C, and no alkalinity (it was assumed that any carbonate scaling risk would be mitigated by acidification to pH 3 with subsequent air stripping of CO_2_).

### 2.3. Plant Modeling

The ZOD technology was simulated using dedicated software written by the authors in C programming language; see the source code [21]. The empirical correlations and border conditions used by the software were based on the indicators provided by the company that operates the plant and were previously discussed in [23]. See Appendix A for the equations used in the modeling. The calculation algorithm was as follows:

1. Calculate the composition of the feed water using ‘Ziemowit-650’ and the required variation in Cl^−^/Mg^2+^/Ca^2+^/SO_4_^2−^ concentration (from −10% to +10%), change the Na^+^ concentration to ensure the electroneutrality condition is preserved;
2. If nanofiltration is included, calculate the permeate composition assuming the ion rejection coefficient obtained in batch-mode experiments, permeate recovery of 74.3%, and the ionic composition obtained in Step 2. Calculate the NF energy consumption [23], assuming 1 m^3^ of feed, 74.3% permeate recovery, and 40 bar operating pressure;
3. Using mass balance equations, calculate the composition of the process streams in the evaporator step, assuming the final Cl^−^ concentration in the concentrate as 176 kg/m^3^ [23] and 1 m^3^ of ‘Ziemowit-650’ or 0.743 m^3^ of NF permeate as evaporator feed;
4. Knowing the amount of water that must be evaporated, calculate the energy consumption in the evaporator, assuming a specific energy consumption of 44 kWh/m^3^ of distillate. This is the empirical value of the electric energy consumption of the vapor compression unit, part of the ZOD technology implemented in the Czerwionka-Leszczyny salt production plant. While it should be possible to decrease the energy consumption of the evaporator by applying modern more efficient technology or by utilizing waste heat where available, we have chosen to stick to the current industrial practice and decided that redesigning the thermal part of the technology is beyond the scope of this paper;
5. Minimize the error function of the crystallizer using the mass balance equations. The amount of evaporated water, crystallized salt, and gypsum are independent variables, while the maximum chloride concentration after crystallization (200 g/dm^3^), the value of the gypsum solubility product (4.302 × 10^−6^), and the maximum concentration of bivalent cations as their respective chlorides in the post-crystallization lyes (8% *w*/*w*) are the boundary conditions;
6. Calculate the energy consumption of the crystallizer, assuming the specific energy consumption of 66 kWh/m^3^ of the distillate.

The values of the specific energy consumption and the system boundary were based on the empirical data of the ZOD technology obtained from the operating company.

### 2.4. Economic Model

CAPEX analysis of the nanofiltration was performed using the BrineTechTools open-source Python library developed by the German Aerospace Center (DLR) [24]. The software was modified to fit the investigated case by changing the NF rejection coefficients, applied pressure, and feed water composition to the ones observed during the batch-mode study of the ‘Ziemowit-650’ coal mine water. The CAPEX of desalination plants depends on the capacity. Since the ZOD technology processes a feed salt load of 10,624 kg/h from the Budryk coal mine currently, it was assumed that if the ‘Ziemowit-650’ coal mine water was to be used as an alternative feed, the salt load would be the same, meaning an NF capacity of 128 m^3^/h was assumed as the basis for CAPEX calculation. Table 3 presents the general economic parameters of the CAPEX model.

## 3. Results and Discussion

### 3.1. Batch-Mode Nanofiltration

Table 4 presents the ionic composition of permeate and retentate samples collected during batch-mode experiments, as well as the averaged value of the permeate, i.e., the cumulative permeate when running the NF from 0% to 74.3% recovery. Based on the averaged permeate, the NF ion rejection coefficients were calculated as Cl^−^ 8.69%, Ca^2+^ 70.8%, Mg^2+^ 85.3%, and SO_4_^2−^ 96.5%. The results show that nanofiltration can successfully remove bivalent impurities from the feed water, which is desirable from the point of view of ZOD technology, as calcium and magnesium limit salt production in the crystallizer used in this technology. Because the NF membrane used shows a lower Ca^2+^ rejection coefficient than the Mg^2+^ rejection coefficient, the generated NF retentate could be a better feedstock for the recovery of Mg(OH)_2_ than raw feed water. The possibility of magnesium recovery from desalination retentates has been extensively studied in the literature [25]. Nanofiltration slightly improves the molar ratio of Ca^2+^ to SO_4_^2−^: from 1.6:1 in feed water to 1.5:1 in NF retentate, which could be beneficial if gypsum is removed from retentate by precipitation before retentate recycling (a solution described in [21,23]), although the effect is too small to be very significant.

### 3.2. Scaling Risk Assessment

Although no scaling was observed during the bench-scale experiments, the possible risk of scaling in large-scale nanofiltration modules should not be neglected. The lack of scaling during the laboratory tests could be explained by a high mixing rate (1200 rpm) in the very simple geometry of the module (just a cylinder). Standard spiral-wound modules used in an industrial-scale nanofiltration plant contain a lot of dead zones where a supersatured solution may stay for a time long enough for the precipitation to happen, something that would not have happened in the bench-scale tests. The obtained retentate composition was used to assess this risk.

Table 5 presents the saturation indices calculated using the Pitzer model with PHREEQC software. The saturation index (SI) is defined as the log10 of the ratio of ion activity product to an equilibrium constant, whereas the saturation level is defined as (10^SI^)·100%. The results show that two sparingly soluble calcium salts are supersaturated: anhydrite (SI of 0.42, which corresponds to a saturation level of 263%) and gypsum (SI of 0.7, saturation level of 501%). Previous research has shown that due to the wide metastable zone of calcium sulfate salts, nanofiltration can be operated safely at saturation levels as high as 500–600% [21] if the hydraulic residence time and the residence time variance in the NF module are kept small enough to ensure that the supersaturated solution leaves the retentate channel before macroscopic crystallization can be observed. The barite scaling in membrane systems at room temperature is manageable at saturation of 460% [26], meaning the NF does not exceed the safe limit. Because there is a possibility of celestite precipitation, the application of anti-scalants might be required. According to He et al. [27], at celestite saturation level of 226–241% in 1-molal sodium chloride solution, addition of 10 mg/dm^3^ of 1-hydroxyethylidene-1,1-diphosphonic acid (HEDP) can prolong nucleation induction time of this salt to 1862 s–125 s, respectively; however, the celestite scaling prevention in nanofiltration requires further investigation and optimization.

### 3.3. Plant Modeling

Table 6 presents the estimated performance of ZOD technology, with and without NF pretreatment. Salt recovery is low in both cases: although coal mine water contains 80.6 kg/m^3^ as NaCl, only 51–58% is recovered as a marketable product. This is a fundamental limitation of ZOD technology, and while NF pretreatment somewhat increases salt recovery, further increases would require replacing the old ZOD system with a new technology, such as those proposed in different studies [28]. However, the objective of this study was to focus specifically on implementing a better pretreatment because replacing the evaporator and crystallizer would incur significant costs and is beyond the current capabilities of the company operating the plant. However, it is clear that the application of NF pretreatment improves the efficiency of the entire process. Because bivalent cations are largely removed from the ZOD feed, the crystallizer used in this technology can reach high recovery before hitting the CaCl_2_+MgCl_2_ limit in post-crystallization lyes. A secondary benefit of NF pretreatment is that NF retentate contains a high load of calcium and magnesium, so it may be used in mineral recovery.

The application of NF pretreatment decreases overall energy consumption by 22%, from 1023 to 791 kWh/t of the produced salt. Nanofiltration decreases the volume of brine treated by ZOD technology, which means that there is less water to evaporate but because the chloride rejection coefficient exhibited by the tested membrane is very low (8.69%), the salt load is not significantly affected. While 1 m^3^ of the ‘Ziemowit-650’ coal mine water contains 80.6 kg of sodium chloride, the 0.743 m^3^ of NF permeate still contains 54.67 kg of sodium chloride, which means only 32% of the initial salt load ends up in the NF retentate. This could be improved by removing gypsum and magnesium hydroxide from the NF retentate and reusing the purified stream in salt production.

One of the important issues to be addressed when designing a desalination plant is how sensitive the proposed solution is to random fluctuations in feed composition. Although the composition of the coal mine waters is generally stable and, unlike surface waters, does not show much seasonal variation, there is still some variability. In this study, it was assumed that each of the major ions under consideration (Cl^−^, Mg^2+^, Ca^2+^, and SO_4_^2−^) can vary up to 10% from the average composition of the ‘Ziemowit-650’ coal mine water. It was also assumed that the change in any of these ions would be accompanied by a change in sodium cation concentration so that the electroneutrality principle is preserved. Based on these assumptions, the effect of feed composition variability on the ZOD energy consumption was estimated, with and without nanofiltration pretreatment.

**Figure 2:**
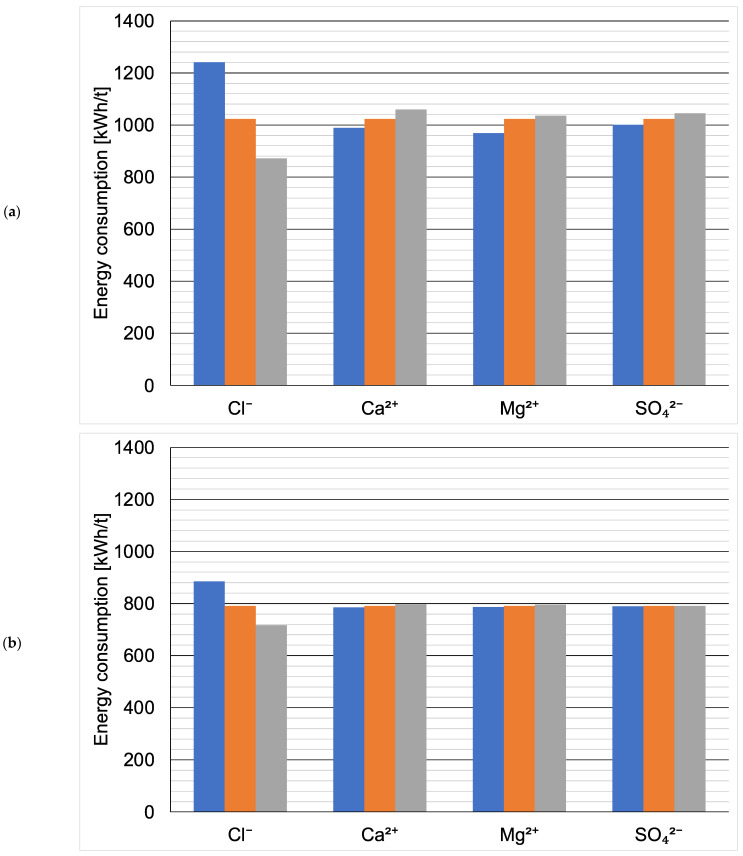
Effect of single-ion concentration variation (blue: −10%, orange: base value, gray: +10%) on projected energy consumption: (**a**) ZOD technology and (**b**) ZOD technology with NF pretreatment.

Figure 2 presents the effect of the fluctuation of the ionic composition on energy consumption in kWh per t of the evaporated salt produced. Chloride concentration has by far the largest effect, which is not surprising considering that sodium chloride is the product here. When ZOD technology is not preceded by nanofiltration, increasing Cl^−^ concentration by 10% decreases the overall energy consumption by 15% (from 1023 kWh/t to 872 kWh/t), as there is less water that needs to evaporate in the evaporator and crystallizer. On the contrary, decreasing the Cl^−^ concentration by 10% increases the energy consumption by 21% (from 1023 kWh/t to 1241 kWh/t). The results show that the ZOD technology is highly sensitive to the sodium chloride concentration of the feed. When nanofiltration pretreatment is applied, the system becomes less sensitive to Cl^−^ variation: increasing the Cl^−^ concentration by 10% decreases the energy consumption by 9.4% (from 791 to 716 kWh/t), while decreasing the Cl^−^ concentration by 10% increases the energy consumption by only 12% (from 791 to 885 kWh/t).

Calcium, magnesium, and sulfate ions have the opposite effect: their presence increases the overall energy consumption of the ZOD technology. This is because the applied crystallizer is sensitive to bivalent cations; there is a practical limit of CaCl_2_ + MgCl_2_ < 8% (*w*/*w*) in post-crystallization lyes. The application of NF pretreatment makes the effect of bivalent-ion fluctuations virtually negligible. This is an important development from a technological point of view: currently, the company operating the plant adds rock salt to the evaporator to decrease the costs and change the ratio of sodium chloride to calcium/magnesium chloride. The application of nanofiltration pretreatment would stabilize the ZOD operation, making it easier to operate and plan.

**Figure 3:**
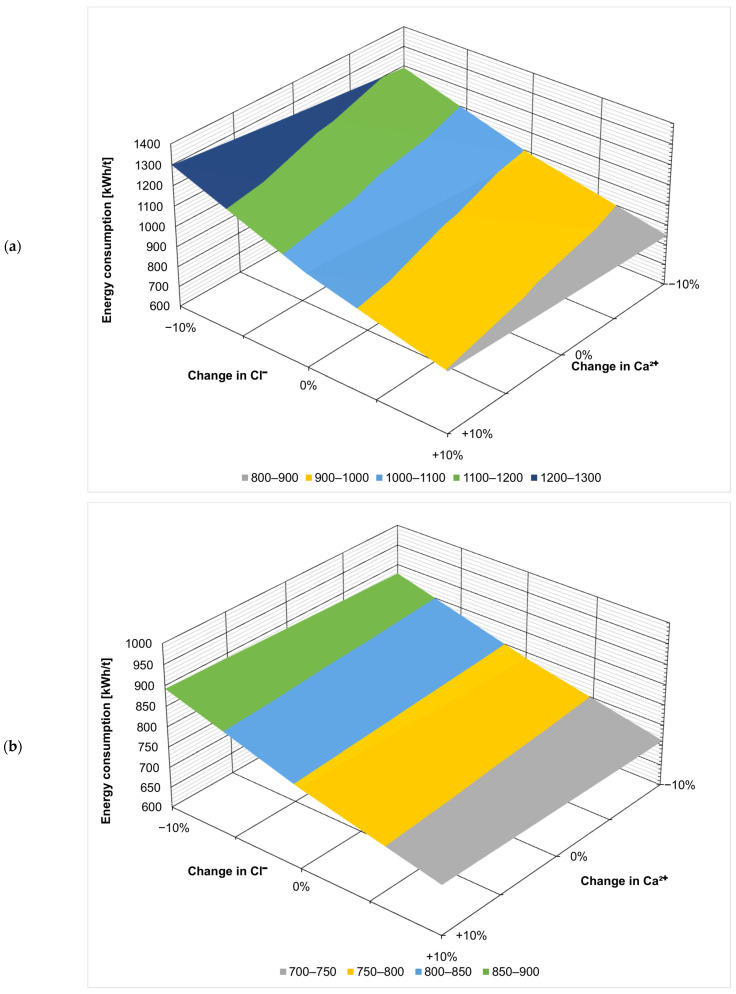
Effect of Cl^−^:Ca^2+^ variation on projected energy consumption: (**a**) ZOD technology and (**b**) ZOD technology with NF pretreatment.

**Figure 4:**
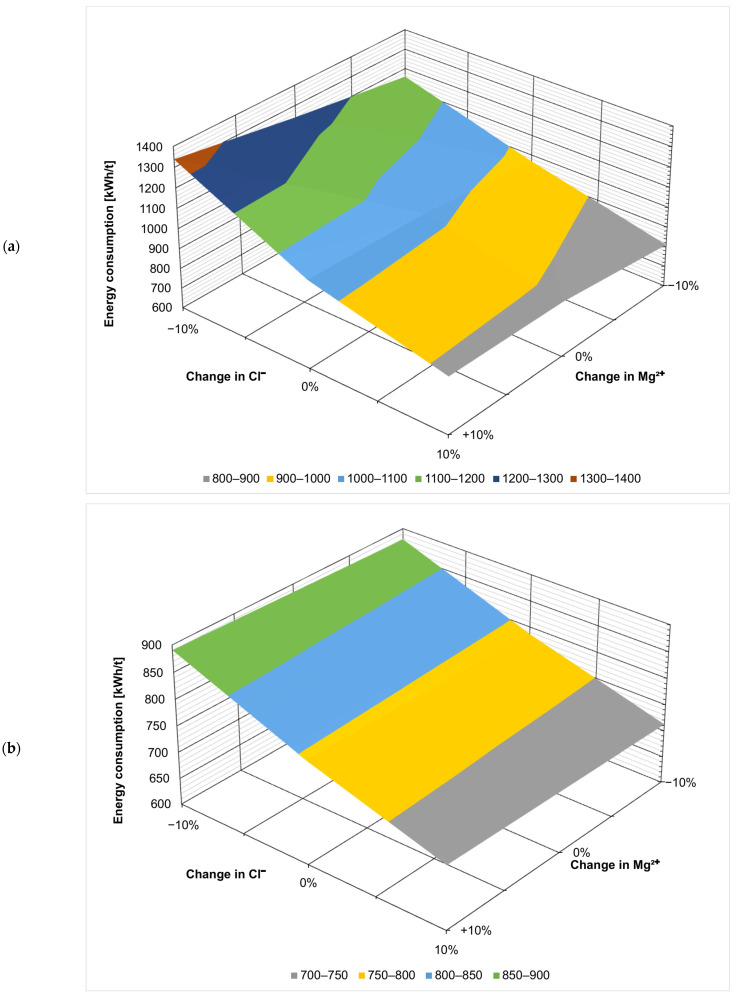
Effect of Cl^−^:Mg^2+^ variation on projected energy consumption: (**a**) ZOD technology and (**b**) ZOD technology with NF pretreatment.

**Figure 5:**
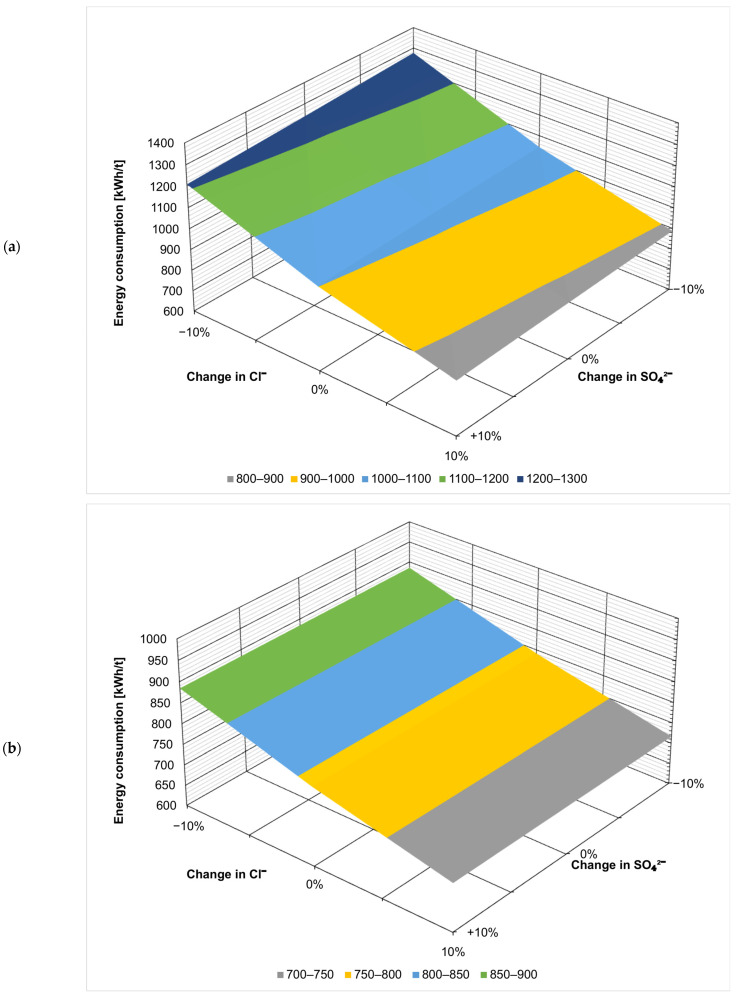
Effect of Cl^−^:SO_4_^2−^ variation on projected energy consumption: (**a**) ZOD technology and (**b**) ZOD technology with NF pretreatment.

**Figure 6:**
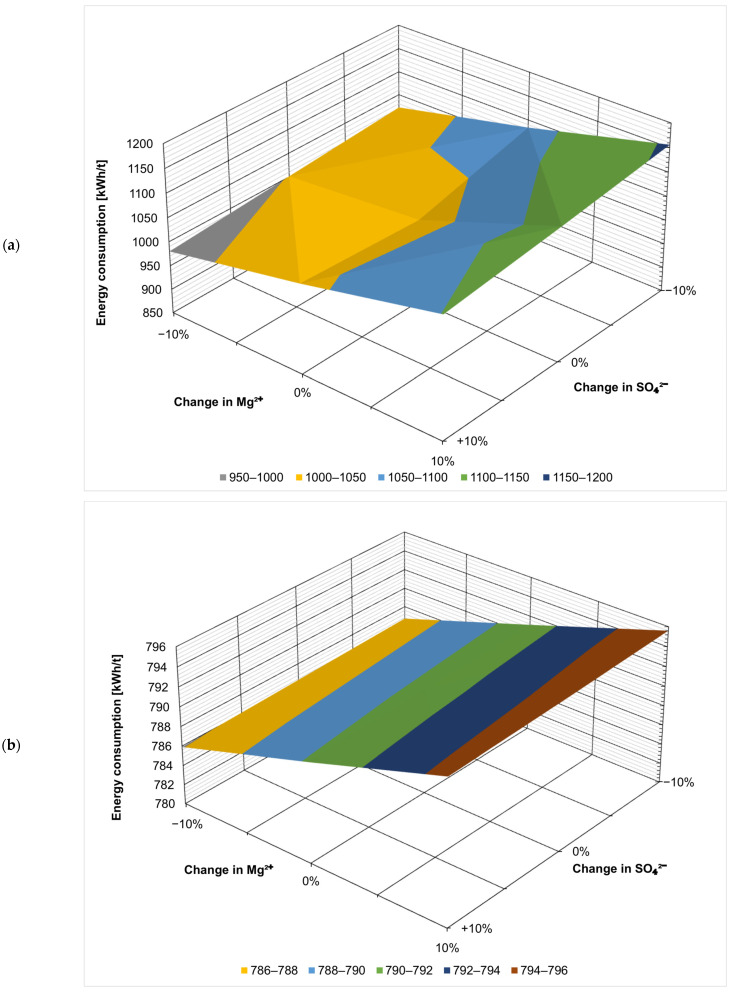
Effect of Mg^2+^:SO_4_^2−^ variation on projected energy consumption: (**a**) ZOD technology and (**b**) ZOD technology with NF pretreatment.

**Figure 7:**
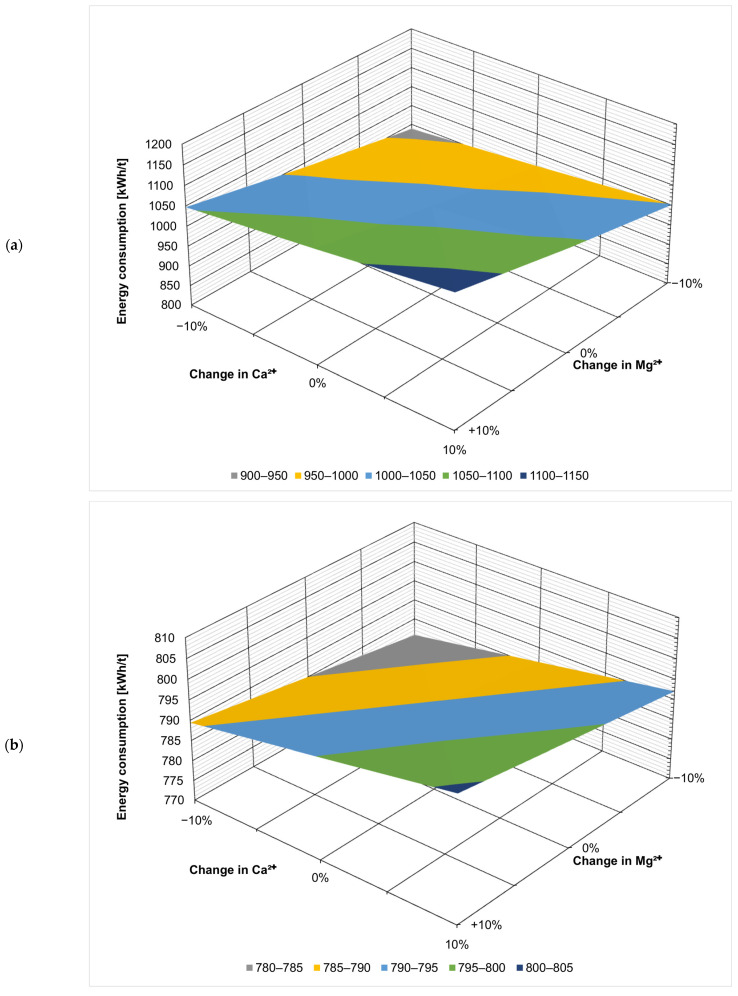
Effect of Mg^2+^:Ca^2+^ variation on projected energy consumption: (**a**) ZOD technology and (**b**) ZOD technology with NF pretreatment.

**Figure 8:**
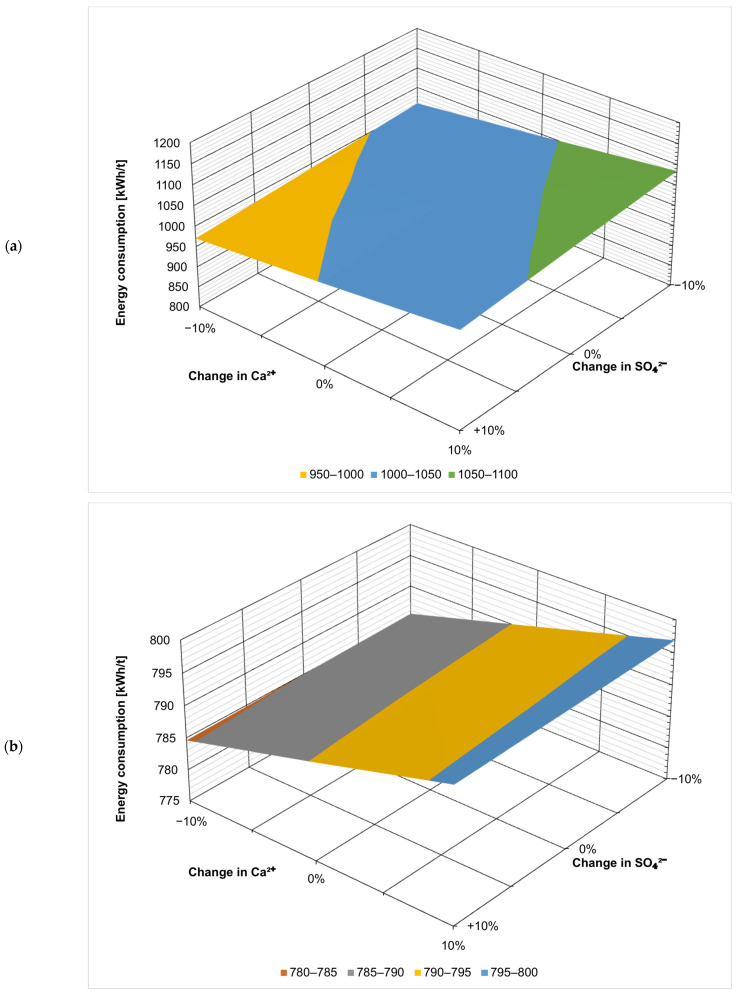
Effect ofCa^2+^:SO_4_^2−^ variation on projected energy consumption: (**a**) ZOD technology and (**b**) ZOD technology with NF pretreatment.

Figure 3, Figure 4, Figure 5, Figure 6, Figure 7 and Figure 8 present the effect of changing multiple ion concentrations on energy consumption. The application of nanofiltration pretreatment significantly dampens the effects the fluctuation of the feed chloride content has on the energy consumption of the ZOD technology. Variations in calcium, magnesium, and sulfates have a negligible effect.

### 3.4. Economic Assessment

The results show that the application of nanofiltration can decrease energy costs and make the ZOD technology more robust for feed concentration variability. However, decreasing energy costs alone is not enough; it must not be offset by the capital and investment costs of building a new unit, otherwise such improvement makes no economic sense. Table 7 presents the result of estimating the CAPEX of the NF plant of 128 m^3^/h capacity, compared with the energy costs generated by ZOD technology of this scale, assuming 0.06 EUR/kWh of electric energy and the price of evaporated, edible salt as 131 EUR/t. The economic assessment of ZOD technology does not take into account the personnel costs, the maintenance of the evaporator/crystallizer, product marketing, etc. In this study, we focus on just one question: can decreased energy consumption and increased salt recovery justify the CAPEX of the NF plant if the ZOD technology were applied to ‘Ziemowit-650’ coal mine water? The results show that the application of NF decreases the annual electric energy costs by 12.4% and increases the income by 13.2%. These two improvements would generate an additional 1,156,207 EUR/y for the company. On the other hand, the cost of the required NF system was estimated at 3,646,294 EUR, with additional NF costs (membrane cleaning chemicals, maintenance, quality control, etc.) of 240,472 EUR/y. This means the initial investment into NF would pay for itself after 4 years, which is still short enough to be an economically viable decision.

A separate issue is NF membrane durability. Working at 40 bar is close to the maximum operating pressure specified by the membrane manufacturer. In this case, the NF worked for only a few hours; in a different, unrelated study [21], the pilot-scale NF unit was operated at high pressure for a few months without experiencing membrane failure. However, typically, NF membranes should last a few years (the CAPEX model used here assumes 5 years), if operating NF at high pressure decreases its lifespan, for example, from 5 years down to 4 years, we simply had no way of testing it. On the other hand, because 40 bar is still below the safe limit indicated by the manufacturer, the question of decreased lifespan was ultimately ignored in the presented calculations.

## 4. Conclusions

The results presented in this work suggest that the application of nanofiltration pretreatment prior to the current evaporative technology (ZOD) could achieve multiple benefits if the water from the ‘Ziemowit-650’ coal mine was used as a new feedstock for salt production to (1) decrease the overall energy consumption by 22%, (2) increase the salt recovery from 51.3% to 58%, and (3) make the ZOD technology less sensitive to random fluctuations in feed water quality. Nanofiltration can be retrofitted to the existing plant and does not require significant downstream changes, which means it can be used as a relatively low-cost and quick improvement in the economy of the current process. However, two issues have to be addressed in future work: the real stability of feed water and the return on investment from using nanofiltration. In this paper, we have arbitrarily chosen a ±10% range of ion concentration changes; however, a long-term sampling of real wastewater would be necessary to establish how the water composition actually fluctuates with time. A separate issue is the economic viability of retrofitting nanofiltration into the already existing plant. A simple economic model used in the paper shows that the decreased energy consumption and increased salt production would offset the CAPEX of the nanofiltration plant after 4 years. However, the ZOD technology is already quite old, as it has been in operation since the early 1990s. The evaporator and crystallizer are already 30 years old and while the company still uses them and, to the best of our knowledge, is not in the process of replacing the old units, it is unclear how many years are ahead of them. The company does not plan to retire ZOD technology in the short term but if it becomes a necessity some years down the road, there might be an opportunity to design a completely new technology, one that does not assume an evaporator-crystallizer system similar to the contemporary one.

## Figures and Tables

**Table 1:** Composition of the coal mine water ‘Ziemowit-650’.

Cl^−^ [g/dm^3^]	Ca^2+^ [g/dm^3^]	Mg^2+^ [g/dm^3^]	SO_4_^2−^ [g/dm^3^]	Na^+^ [g/dm^3^]	Ba [mg/dm^3^]	Sr [mg/dm^3^]
48.91	1.92	2.07	2.85	26.88	<0.1	53.8

**Table 2:** Parameters of the Synder Filtration^TM^ NFW membrane used in the study (source: manufacturer’s data).

Parameter	Value
Polymer	Proprietary PA TFC
Approx. molecular weight cut-off	300–500 Da
MgSO_4_ rejection (test conditions: 2000 ppm MgSO_4_ solution at 7.6 bar operating pressure, 25 °C)	97%
NaCl rejection (test conditions: 2000 ppm NaCl solution at 7.6 bar operating pressure, 25 °C)	20%
Maximum operating pressure at temperature lower than 35 °C	41.37 bar

**Table 3:** General economic parameters of the CAPEX model [24].

Parameter	Value
Plant capacity	128 m^3^/h
Interest rate	6%
Chemical Engineering Plant Cost Index (CEPCI)	576.7
Depreciation of civil costs	30 years
Depreciation of mechanical/electrical costs	15 years
Depreciation of membrane costs	5 years
Cost of a single membrane module	1000 EUR
Plant availability	94%
Electric energy cost	0.06 EUR/kWh
Energy efficiency	80%
Chemicals cost	0.023 EUR/m^3^ of NF permeate

**Table 4:** Ionic composition of the permeate and retentate during the batch mode experiments.

Sample	Permeate Recovery [%]	Concentration [g/dm^3^]			
		Cl^−^	Ca^2+^	Mg^2+^	SO_4_^2−^
Permeate 1	10	37.57	0.28	0.27	-
Permeate 2	20	39.34	0.20	0.19	-
Permeate 3	31.3	40.05	0.36	0.12	-
Permeate 4	40	45.36	0.40	0.17	-
Permeate 5	50	45.01	0.48	0.19	-
Permeate 6	60	47.14	0.68	0.27	-
Permeate 7	70	50.32	1.04	0.39	-
Permeate 8	74.3	52.45	1.04	0.83	-
Retentate	74.3	70.88	6.41	6.81	10.25
Averaged permeate		44.65	0.56	0.30	0.099

**Table 5:** Saturation indices of sparingly soluble salts present in the NF retentate.

Species	Formula	Saturation Index (SI)	Saturation Level [%]
Anhydrite	CaSO_4_	0.42	263
Barite	BaSO_4_	0.54	347
Bischofite	MgCl_2_·6H_2_O	−5.36	0
Bloedite	Na_2_Mg(SO_4_)_2_·4H_2_O	−3.69	0
Brucite	Mg(OH)_2_	−11.96	0
Celestite	SrSO_4_	0.37	234
Epsomite	MgSO_4_·7H_2_O	−1.87	1
Glauberite	Na_2_Ca(SO_4_)_2_	−0.9	13
Gypsum	CaSO_4_·2H_2_O	0.7	501
H_2_O_(g)_	H_2_O	−1.54	3
Halite	NaCl	−1.34	5
Hexahydrite	MgSO_4_·6H_2_O	−2.11	1
Kieserite	MgSO_4_·H_2_O	−3.23	0
Labile S	Na_4_Ca(SO_4_)_3_·2H_2_O	−3.08	0
Leonhardite	MgSO_4_·4H_2_O	−2.72	0
Magnesium chloride dihydrate	MgCl_2_·2H_2_O	−15.18	0
Magnesium chloride tetrahydrate	MgCl_2_·4H_2_O	−7.67	0
Mirabilite	Na_2_SO_4_·10H_2_O	−1.55	3
Pentahydrite	MgSO_4_·5H_2_O	−2.36	0
Portlandite	Ca(OH)_2_	−18.01	0
Thenardite	Na_2_SO_4_	−2.13	1

**Table 6:** Process performance in each of the simulated cases.

Parameter		ZOD Technology	ZOD Technology with NF Pretreatment
Energy consumption [kWh/t of salt produced]	Nanofiltration	0	37
	Evaporator	769	522
	Crystallizer	254	232
	Total	1023	791
Mass of salt produced salt [kg/m^3^ of coal mine water]		41.31	46.77
Gypsum mass produced by crystallizer [kg/m^3^ of coal mine water]		4.850	0.111
Salt recovery [%]		51.3	58.0
Volume of postcrystallisation lyes [m^3^/m^3^ of coal mine water]		0.119	0.024
Volume of NF retentate [m^3^/m^3^ of coal mine water]		0	0.257

**Table 7:** Economic assessment of the ZOD and NF-ZOD cases for a 128 m^3^/h plant working on ‘Ziemowit-650’ coal mine water.

Parameter	ZOD Technology	ZOD Technology with NF Pretreatment
Mass of salt produced [t/y]	46,320 *	52,442 *
Electric energy costs [EUR/y]	2,843,122	2,488,897
Income from selling salt [EUR/y]	6,067,920	6,869,902
CAPEX of NF [EUR]	0	3,646,294
NF chemicals costs [EUR/y]	0	21,694
NF maintenance costs [EUR/y]	0	72,926
NF other costs excluding energy [EUR/y]	0	145,852

* the company operating ZOD technology mixes coal mine water with rock salt to increase the production, the value given here represents only the salt obtained purely from coal mine water assuming no rock salt would be added if ‘Ziemowit-650’ were to be the new feed water.

## Data Availability

The original contributions presented in the study are included in the article, further inquiries can be directed to the corresponding author.

## Appendix A

The ionic composition of the feed water going into the system was calculated by varying concentrations of ions present in the ‘Ziemowit-650’ coal mine (48.91 kg/m^3^ as Cl^−^, 1.92 kg/m^3^ as Ca^2+^, 2.07 kg/m^3^ as Mg^2+^, 2.85 as SO_4_^2−^) water by −10%, 0%, or +10%.

C_f,Cl_^−^ = 48.91 × (100% ± 10%), (A1)

C_f,Ca_^2+^ = 1.92 × (100% ± 10%), (A2)

C_f,Mg_^2+^ = 2.07 × (100% ± 10%), (A3)

C_f,SO4_^2−^ = 48.91 × (100% ± 10%), (A4)

where C_f,Cl_^−^, C_f,Ca_^2+^, C_f,Mg_^2+^, and C_f,SO4_^2−^ are the concentration of chlorides, calcium, magnesium, and sulfates, respectively, in the feed water going into the system. Sodium concentration in the feed, C_Na_^+^, was calculated based on the electroneutrality condition

C_f,Na_^+^ = (C_f,Cl_^−^/35.5 + 2 × C_f,SO4_^2−^/96 − 2 × C_f,Ca_^2+^/40 − 2 × C_f,Mg_^2+^/24) × 23, (A5)

In the case of the ZOD system with NF pretreatment, the ionic composition of nanofiltration permeate was calculated based on rejection coefficients of chloride, calcium, magnesium, and sulfate (R_Cl_^−^, R_Ca_^2+^, R_Mg_^2+^, and R_SO4_^2−^, respectively) obtained in the batch-mode study and the electroneutrality condition for sodium:

C_NF,Cl_^−^ = C_f,Cl_^−^ × R_Cl_^−^, (A6)

C_NF,Ca_^2+^ = C_f,Ca_^2+^ × R_Ca_^2+^, (A7)

C_NF,Mg_^2+^ = C_f,Mg_^2+^ × R_Mg_^2+^, (A8)

C_NF,SO4_^2−^ = C_f,SO4_^2−^ × R_SO4_^2−^, (A9)

C_NF,Na+_ = (C_NF,Cl_^−^/35.5 + 2 × C_NF,SO4_^2−^/96 − 2 × C_NF,Ca_^2+^/40 − 2 × C_NF,Mg_^2+^/24) × 23, (A10)

where C_NF,Cl_^−^, C_NF,Ca_^2+^, C_NF,Mg_^2+^, C_NF,SO4_^2−^, and C_NF,Na+_ are the concentration of chlorides, calcium, magnesium, sulfates, and sodium, respectively, in the nanofiltration permeate. The volume of the NF permeate, V_NF_, and the volume of NF retentate, V_R_, were calculated using the volume of the feed water, V_f_ = 1 m^3^, and NF permeates recovery, Y = 74.3%.

V_NF_ = Y × V_f_, (A11)

V_R_ = V_f_ − V_NF_, (A12)

To calculate the energy consumption of the nanofiltration in kWh, E_NF_, at pressure P of 40 bar, the following semi-empirical equation explained in previous work [28] was used

E_NF_ = V_NF_·[0.05 + 0.03244 × P/Y − 0.02695 × P × (1 − Y)/Y], (A13)

In the case of calculating the ZOD system without the NF pretreatment, Eqns. A6-A13 can be skipped, instead assuming V_NF_ = V_f_, V_R_ = 0, C_NF,Cl_^−^ = C_f,Cl_^−^, C_NF,Ca_^2+^ = C_f,Ca_^2+^, C_NF,Mg_^2+^ = C_f,Mg_^2+^, C_NF,SO4_^2−^ = C_f,SO4_^2−^, C_NF,Na+_ = C_f,Na+_, Y = 100%, and E_NF_ = 0; in other words, the theoretical NF permeate has the same ionic composition and volume as the feed and the energy consumption of NF is omitted.

Next, to calculate the evaporator of the ZOD technology, it was assumed that the maximum chloride concentration in the evaporator concentrate, C_VC,Cl_^−^, is 176 kg/m^3^. The concentration of the calcium, magnesium, sulfates, and sodium in the evaporator concentrate (C_VC,Ca_^2+^, C_VC,Mg_^2+^, C_VC,SO4_^2−^, and C_VC,Na+_, respectively), as well as the volume of the evaporator concentrate, V_VC_, were calculated based on mass balances, assuming the evaporator distillate has no salinity

C_VC,Cl_^−^ = 176, (A14)

V_VC_ = C_NF,Cl_^−^ × V_NF_/C_VC,Cl_^−^, (A15)

C_VC,Ca_^2+^ = C_NF,Ca_^2+^ × V_NF_/V_VC_, (A16)

C_VC,Mg_^2+^ = _CNF,Mg_^2+^ × V_NF_/V_VC_, (A17)

C_VC,SO4_^2−^ = C_NF,SO4_^2−^ × V_NF_/V_VC_, (A18)

C_VC,Na+_ = C_NF,Na+_ × V_NF_/V_VC_, (A19)

The ZOD evaporator consumes 44 kWh per 1 m^3^ of distillate; therefore, the energy consumption of the evaporator in kWh, EVC, is

E_VC_ = 44 × (V_NF_ − V_VC_), (A20)

The mass balances of the ZOD crystallizer were as follows

C_RCC,Cl_^−^ × V_RCC_ + m_s_/58.5 × 35.5 = C_VC,Cl_^−^ × V_VC_, (A21)

C_RCC,Ca_^2+^ × V_RCC_ + m_g_/172 × 40 = C_VC,Ca_^2+^ × V_VC_, (A22)

C_RCC,Mg_^2+^ × V_RCC_ = C_VC,Mg_^2+^ × V_VC_, (A23)

C_RCC,SO4_^2−^ × V_RCC_ + m_g_/172 × 96 = C_VC,Cl_^−^ × V_VC_, (A24)

C_RCC,Na+_ × V_RCC_ + m_s_/58.5 × 23 = C_VC,Na+_ × V_VC_, (A25)

d_RCC_ × V_RCC_ + m_w_ = d_VC_ × V_VC_, (A26)

where C_RCC,Cl_^−^, C_RCC,Ca_^2+^, C_RCC,Mg_^2+^, C_RCC,SO4_^2−^, and C_RCC,Na+_ are the concentration of, respectively, chlorides, calcium, magnesium, sulfates, and sodium in the post-crystallization lyes; m_s_ is the mass of edible salt (NaCl) produced; m_g_ is the mass of the gypsum (CaSO_4_·2H_2_O) produced; V_RCC_ is the volume of the post-crystallization lyes; m_w_ is the mass of water evaporated by the crystallizer; and d_RCC_ and d_VC_ are the density of post-crystallization lyes and evaporator concentrate, respectively, calculated based on empirical equations

d_RCC_ = 7.7526 × [(C_RCC,Cl_^−^ × 58.5/35.5)/(d_VC_ × V_VC_)] × 100 + 997.05, (A27)

d_VC_ = s^4^ × 9.248 × 10^−10^ − s^3^ × 2.834 × 10^−7^ − s^2^ × 2.26518 × 10^−4^ + s × 0.6798978 + 1004.0208, (A28)

s = (C_VC,Cl_^−^ × 58.5/35.5), (A29)

To calculate the m_s_, m_g_, and m_w_, the error function ERF, a sum of squared errors related to chloride solubility, bivalent contaminants limit, and the gypsum solubility were minimized using Nelder–Mead optimization algorithm and m_s_, m_g_, and m_w_ as independent variables

ERF = (ERR_1_)^2^ + (ERR_2_)^2^ + (ERR_3_)^2^, (A30)

ERR_1_ = (200 − C_RCC,Cl_^−^)/200, (A31)

ERR_2_ = [0.08 − 111 × C_RCC,Ca_^2+^/(V_RCC_ × d_RCC_ × 40) − 95 × C_RCC,Mg_^2+^/(V_RCC_ × d_RCC_ × 24)]/0.08, (A32)

ERR_3_ = [4.30165423622131 × 10^−6^ − (C_RCC,Ca_^2+^ × C_RCC,SO4_^2−^)^2^]/(4.30165423622131 × 10^−6^), (A33)

The ZOD crystallizer consumes 66 kWh of electric energy per 1 m^3^ of evaporated distillate, so the energy consumption of the crystallizer in kWh, E_RCC_, can be calculated as

E_RCC_ = 66 × (V_VC_ − V_RCC_), (A34)
